# Alternative Fixation of a Comminuted Fibular Head Avulsion in a Patient With an Ipsilateral Tibial Intramedullary Nail: A Hybrid Construct Case Report

**DOI:** 10.7759/cureus.117027

**Published:** 2026-09-27

**Authors:** Kannan Murthy, Tiffany T Nguyen, Akshay Som, Youssef Messiha, Najib Ussef

**Affiliations:** 1 Orthopedics, Kern Medical Center, Bakersfield, USA; 2 Surgery, Bakersfield Memorial Hospital, Bakersfield, USA; 3 Orthopedics, Western University of Health Sciences, Pomona, USA; 4 Orthopedic Surgery, Kern Medical Center, Bakersfield, USA

**Keywords:** common peroneal neurolysis, fibular head avulsion, hook plate fixation, krackow suture, lateral collateral ligament repair, posterolateral corner injury, retained hardware, tibial intramedullary nail

## Abstract

Fibular head avulsion fractures are uncommon injuries that disrupt the posterolateral corner of the knee and are frequently accompanied by injury to the adjacent common peroneal nerve. Current fixation options, cannulated screw fixation, locking compression hook plates, and suture anchor or transosseous repair, generally require either a fragment large enough to accept a screw or an intact, unobstructed proximal fibula for tunnel placement. We report a 33-year-old male pedestrian struck by a motor vehicle. He had an open proximal tibial fracture treated with intramedullary nailing and, approximately one week later, was found to have a comminuted fibular head avulsion. He also sustained posterolateral corner disruption, common peroneal nerve palsy, and a positive Lachman exam. The retained tibial intramedullary nail constrained conventional screw trajectories and bone-tunnel placement, while comminution of the avulsion fragment hindered reliable single-implant fixation. The fragment was stabilized with a hybrid construct combining transosseous Krackow sutures of FiberWire through the biceps femoris tendon, passed via Beath pins and tied over the anteromedial tibia. Additionally, a three-hole 3.5 mm locking compression plate (LCP) hook plate was applied to buttress the proximal fibula, along with primary repair of a mid-substance LCL tear, and formal common peroneal neurolysis. Examination under anesthesia after fixation demonstrated reduction of varus stress gapping from +3 to neutral. At nine weeks, the patient had knee range of motion of 0° to 115°, had advanced to weight-bearing as tolerated, and demonstrated maintained reduction with intact hardware and interval healing on radiographs. The common peroneal nerve palsy subsequently resolved completely; given full clinical recovery, electromyography was not indicated. This case describes a reproducible alternative technique for fibular head avulsion complicated by comminution and retained intramedullary hardware.

## Introduction

The proximal fibula serves as the attachment site for several structures of the posterolateral corner (PLC), including the fibular collateral ligament (FCL, also known as LCL), biceps femoris tendon, popliteofibular ligament, and the arcuate ligament complex [1]. The PLC functions as a primary restraint against varus stress, external tibial rotation, and posterolateral rotatory instability, making preservation of these attachments essential to maintaining knee stability [1,2]. Avulsion fracture of the fibular head, recognized radiographically as the “arcuate sign,” is an important marker of PLC injury and may be associated with injury to the common peroneal nerve, which courses immediately posterolateral to the fibular neck [3,4]. If inadequately treated, disruption of these stabilizing structures may result in chronic instability, persistent pain, and functional limitation [5,6]. The proximity of the common peroneal nerve further complicates surgical management, requiring careful identification and protection during fixation.

Several surgical fixation strategies have been described for fibular head avulsion fractures, including cannulated screw fixation of large fragments [7], locking compression hook plates [8], and suture-anchor or transosseous suture repair of the avulsed soft-tissue attachments [9]. However, these techniques generally require either a sufficiently large fracture fragment for screw fixation or an unobstructed proximal fibula for bone-tunnel replacement [10]. To our knowledge, no published technique addresses the combination of a comminuted fibular head avulsion fracture and an ipsilateral tibial intramedullary nail. In this setting, the retained implant may restrict screw trajectory and transosseous tunnel placement, while fracture comminution limits the ability to achieve stable fixation with a single implant.

We present a hybrid fixation construct developed to address the combined challenges of fracture comminution and retained ipsilateral tibial hardware, with concurrent management of the associated common peroneal nerve injury. We also discuss the rationale and reproducibility of this technique.

## Case presentation

History and presentation

A 33-year-old male was struck by a motor vehicle as a pedestrian and sustained an open proximal third tibial fracture with an associated left fibular head avulsion and left common peroneal nerve palsy. The tibial fracture was addressed emergently with intramedullary nailing (DePuy Synthes, West Chester, PA, USA) due to its open nature. Approximately one week postoperatively, attention was turned to the fibular head avulsion. Associated injuries per the discharge summary included a left first rib fracture, a sacral fracture, a pubic root fracture with bilateral superior pubic rami fractures, and two metallic foreign bodies (2.4 cm and 2.0 cm) within the stomach and gastrointestinal tract.

Examination

On initial presentation, neurovascular examination of the left lower extremity demonstrated intact dorsiflexion and plantar flexion of the ankle and toes, 2+ dorsalis pedis and posterior tibial pulses, capillary refill under two seconds, intact sensation, and soft compartments.

On re-examination approximately one week later, before operative fixation of the fibular head avulsion, the patient was unable to dorsiflex the left ankle, and toe flexion/extension were diminished on the left compared to the right, consistent with a common peroneal nerve palsy that had developed or become clinically apparent since the initial injury. Pulses, capillary refill, and compartments remained unchanged. Examination under anesthesia demonstrated a positive Lachman test and +3 varus stress laxity at both 0° and 30° of knee flexion. The range of motion was 0° to 120°.

Imaging

The initial anteroposterior radiograph of the left knee demonstrated a comminuted, displaced proximal third tibial fracture with an associated fibular head avulsion fracture; the fibular head fragment was displaced laterally and retracted proximally with visible comminution (Figure 1A). The anteroposterior radiograph obtained after emergent tibial intramedullary nailing demonstrated the nail in satisfactory position with proximal interlocking screws traversing the proximal tibial metaphysis. The fibular head avulsion fragment remained displaced, and the proximal interlocking screws occupied the corridor that would conventionally be used for transosseous tunnel placement or cross-screw fixation of the fibular head (Figure 1B).

**Figure 1:**
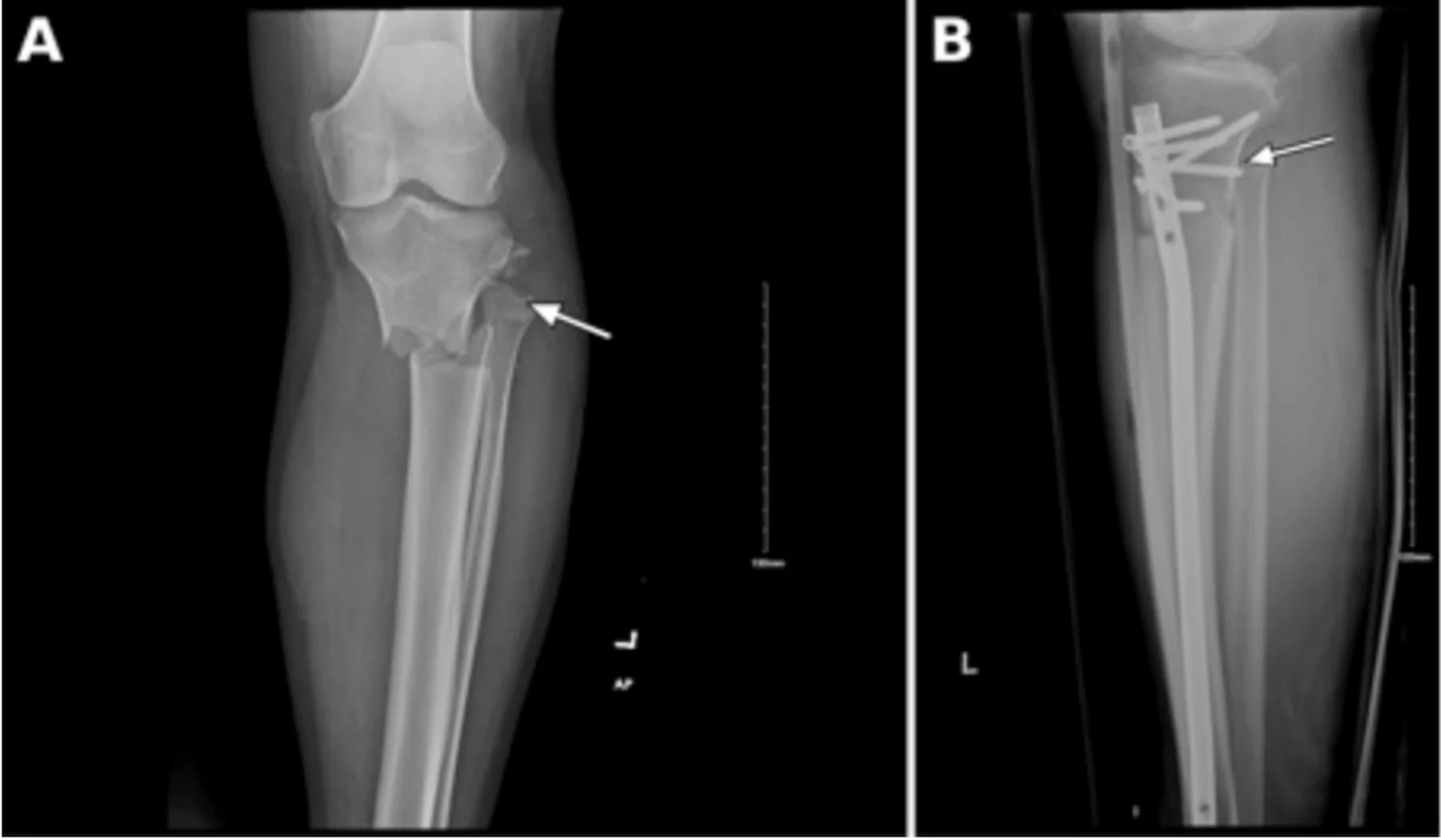
Anteroposterior radiographs of the left knee at presentation and after emergent tibial intramedullary nailing. (A) Initial radiograph demonstrating a comminuted, displaced proximal third tibial fracture with an associated fibular head avulsion fracture; the avulsed fragment (arrow) is displaced laterally and retracted proximally with visible comminution. (B) Radiograph after emergent tibial intramedullary nailing demonstrating the nail in satisfactory position; the proximal interlocking screws (arrow) occupy the corridor conventionally used for transosseous tunnel placement or cross-screw fixation of the fibular head, and the fibular head fragment remains displaced. AP: anteroposterior; L: left laterality marker.

Postoperative anteroposterior and lateral radiographs obtained after hybrid fixation demonstrated the three-hole 3.5 mm locking compression plate (LCP) hook plate (DePuy Synthes, West Chester, PA, USA) with tines engaged in the fibular head fragment and three cortex screws in satisfactory position, the transosseous suture construct, and the tibial intramedullary nail with intact hardware. The fibular head fragment was reduced to a near-anatomic position (Figures 2A, 2B).

**Figure 2:**
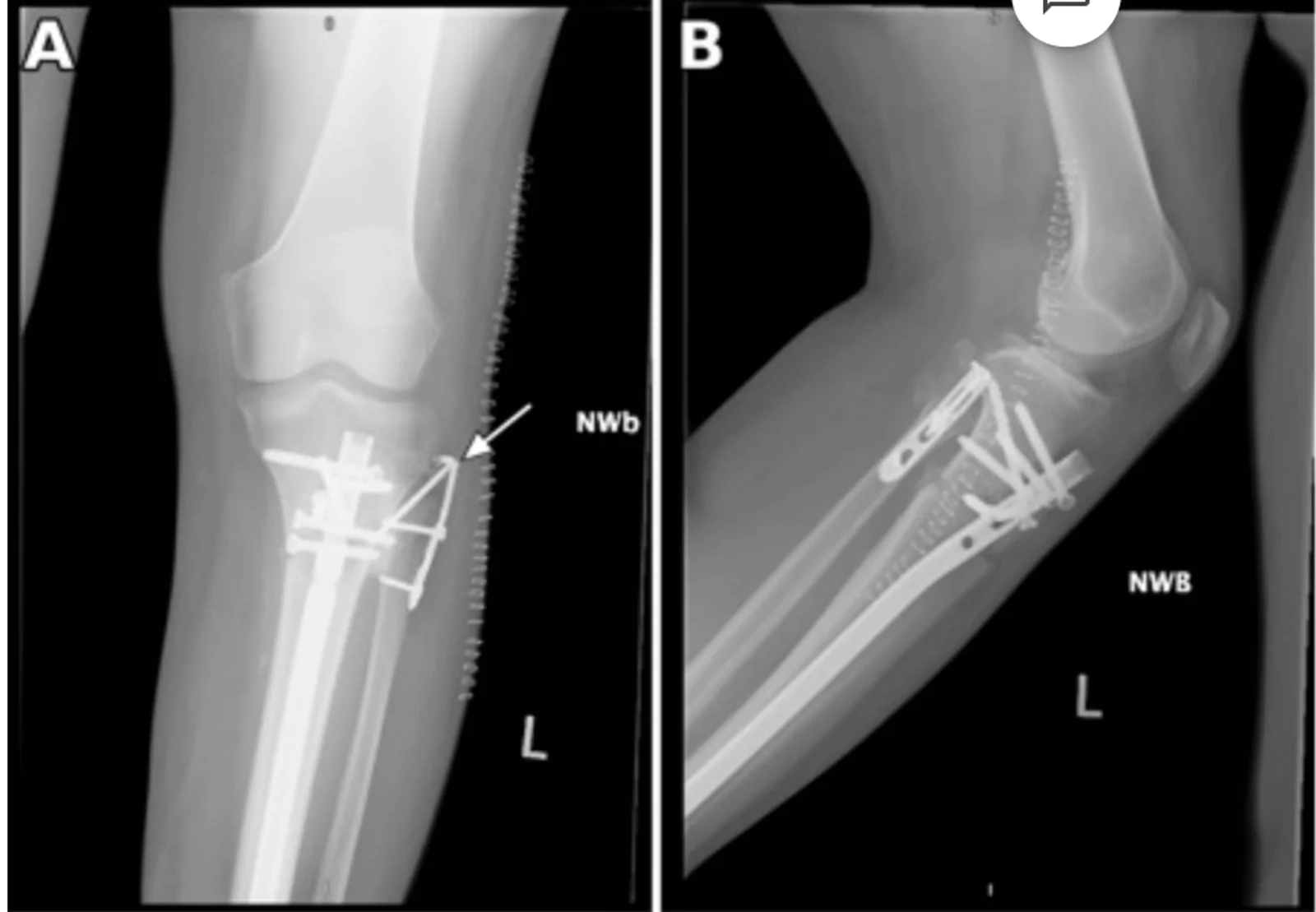
Postoperative anteroposterior and lateral radiographs of the left knee after hybrid fixation of the fibular head avulsion. (A) Anteroposterior radiograph demonstrating the 3.5 mm LCP hook plate (DePuy Synthes, West Chester, PA, USA) with its tines (arrow) engaged in the fibular head fragment and three cortex screws in satisfactory position; the transosseous suture construct and the tibial IMN with intact hardware are also demonstrated, with the fibular head fragment reduced to a near-anatomic position. (B) Lateral radiograph demonstrating maintained reduction and overall construct position. LCP: locking compression plate; L: left laterality marker; NWB/NWb: non-weight-bearing.

Diagnosis and indication for surgery

The patient was diagnosed with a comminuted left fibular head avulsion fracture with associated posterolateral corner disruption, including a mid-substance LCL tear and +3 varus stress laxity at both 0° and 30° of knee flexion on examination under anesthesia. A positive Lachman test was identified on examination under anesthesia, consistent with an anterior cruciate ligament (ACL) tear; this clinical diagnosis was supported by A/P radiographs demonstrating a tibial spine avulsion fracture. Advanced imaging with MRI was not pursued, as the retained tibial intramedullary nail would produce substantial metallic artifact degrading diagnostic quality. The delay required for MRI scheduling was deemed clinically inappropriate given the need for timely operative management of the PLC injury. Assessment of the remaining PLC structures was limited to clinical examination under anesthesia, which demonstrated the aforementioned varus laxity and mid-substance LCL disruption; the popliteal tendon and popliteofibular ligament could not be individually assessed under the clinical circumstances. A left common peroneal nerve palsy had developed or become clinically apparent following the initial tibial nailing, with the patient unable to dorsiflex the left ankle at the time of re-examination approximately one week postoperatively. Operative fixation was indicated to restore posterolateral corner stability and preserve the bony insertion of the LCL and biceps femoris.

Operative technique

Under general anesthesia, the patient was positioned supine with all bony prominences padded. The left lower extremity was sterilely prepared and draped. A lateral incision was centered over the fibular head and Gerdy's tubercle, and dissection was carried down to the fibular head fracture fragment, which was significantly displaced and retracted proximally with comminution. Callus was debrided from the fracture site.

A formal common peroneal nerve neurolysis was performed around the fibular neck and extended proximally to evaluate the integrity of the nerve. The nerve was found to be in continuity but exhibited significant swelling and edema consistent with a stretch/traction injury versus direct contusion. The mid-substance tear of the LCL was identified and repaired primarily using 0 Vicryl suture (Ethicon, Somerville, NJ, USA) in a figure-of-eight fashion. Primary repair with absorbable suture was selected given the acute presentation, adequate tissue quality at the tear site, and the concurrent bony fixation of the fibular head restoring the distal LCL attachment. The use of absorbable suture in this acute setting was considered acceptable, as the repaired ligament was protected postoperatively with a hinged knee brace locked in extension for ambulation, limiting varus stress during the period of healing.

The fibular head fracture fragment was reduced with direct manual pressure combined with Krackow locking sutures [11] placed through the biceps femoris tendon using #2 FiberWire suture (Arthrex, Naples, FL, USA). A Kirschner wire was placed to maintain provisional reduction. The limbs of the Krackow suture were passed through Beath pins directed in a distal anteromedial trajectory through the tibia and tied over the anteromedial cortex through a separate 3 cm incision, re-tensioning the biceps femoris to its fibular insertion while routing the tunnels to avoid the retained intramedullary nail.

Despite the transosseous suture repair, residual gapping persisted at the fracture site. Severe comminution precluded direct screw fixation of the fibular head. Conventional alternatives were also unviable: trans-tibial cannulated screws were blocked by the interlocking screws of the retained tibial nail, and the osteoporotic bone was too frail to support suture anchors against the pull of the biceps femoris and LCL. The common peroneal nerve was carefully elevated with a Freer elevator at the fibular neck, and the hook plate was advanced from proximal to distal directly on bone, taking care to protect the nerve at all times. The hook tines were engaged into the proximal fibular head avulsion fragment, two distal 3.5 mm cortex screws were placed into the fibular shaft to secure the plate (solely engaging the fibula, without crossing into the tibia). An additional 3.5 mm cortex screw was placed in the most proximal plate hole directed in a proximal-to-distal, medially oriented trajectory to avoid the intramedullary nail. Construct placement and reduction were confirmed under direct fluoroscopy, which demonstrated appropriate hardware position and satisfactory fracture reduction (Figure 2). Varus stress testing showed gapping reduced from +3 to neutral.

Vancomycin powder was applied to all surgical wounds. The subcutaneous tissue was closed with 3-0 Vicryl in an interrupted fashion, and skin edges were reapproximated with staples. Estimated blood loss was 50 mL. All counts were correct, and the patient was extubated without complication.

Postoperative course and rehabilitation

The patient was made toe-touch weight-bearing on the left lower extremity and placed in a hinged knee brace locked in extension for ambulation and sleeping, with the brace unlocked during supervised physical therapy for range-of-motion exercises. An ankle-foot orthosis (AFO) was prescribed for the peroneal nerve palsy-associated foot drop. Postoperative antibiotics were administered, and DVT prophylaxis was initiated with aspirin 81 mg twice daily. The patient and family were counseled that a tibialis anterior tendon transfer, ankle arthrodesis, or long-term carbon-fiber AFO use might be necessary if the peroneal nerve palsy did not resolve, and that electromyography would be considered at a future date depending on clinical recovery. The patient was discharged to an ARU after a 16-day hospitalization.

Follow-up and outcome

At the four-week postoperative visit, the patient demonstrated early signs of neurological recovery, with mild but incomplete return of great toe dorsiflexion on the left. Full ankle dorsiflexion had not yet returned, and the patient remained in the ankle-foot orthosis. This represented partial recovery of common peroneal nerve function. At the nine-week visit, the common peroneal nerve palsy had resolved completely, with full motor strength in ankle dorsiflexion and toe extension and intact sensation in the superficial and deep peroneal nerve distributions on clinical examination. Given complete clinical recovery, EMG was no longer indicated. The neurological recovery is attributed to a combination of spontaneous nerve recovery-consistent with the natural history of traction injuries to the common peroneal nerve-and the intraoperative neurolysis. The relative contribution of each cannot be determined from this single case. The peroneal nerve deficit was not documented on the initial ED presentation and was identified on re-exam approximately one week later, prior to fibular head fixation. Whether it developed after tibial nailing or was initially unrecognized in the setting of the patient’s polytrauma cannot be established with certainty.

On clinical examination at the nine-week visit, varus stress testing of the left knee demonstrated neutral (+0) laxity at both 0° and 30° of knee flexion, equal to the contralateral side. Soft tissues were intact; there were no signs of irritation or wound breakdown. The patient was ambulating without difficulty and was satisfied with his functional recovery. These findings represent early clinical stability; however, nine-week follow-up cannot establish definitive ligament healing or long-term biomechanical adequacy, and continued surveillance is needed. Radiographs at nine weeks demonstrated maintained near-anatomic alignment of the fibular head fragment, intact LCP hook plate and cortex screws, and an unchanged tibial intramedullary nail with interval healing changes (Figure 3). The ACL tear was managed nonoperatively in the acute setting, as the retained tibial intramedullary nail precluded tunnel-based ACL reconstruction. The patient was not interested in any further surgery as he remained satisfied with his post-operative course.

**Figure 3:**
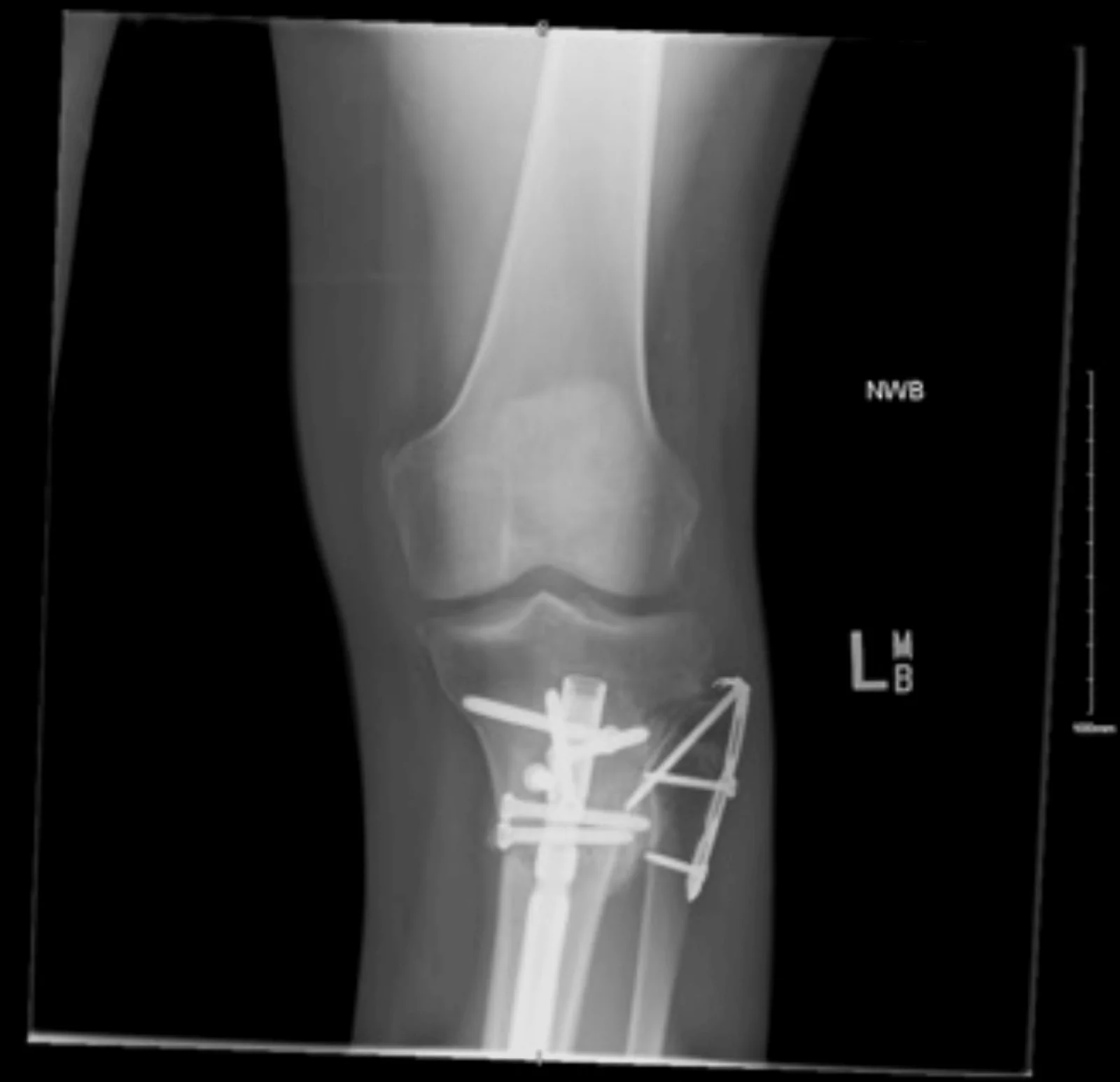
Anteroposterior radiograph of the left knee at nine-week follow-up. The LCP hook plate tines remain engaged in the fibular head fragment, the cortex screws are intact, and the tibial intramedullary nail with proximal interlocking screws is unchanged in position. The fibular head fragment remains in near-anatomic alignment. L: left laterality marker; NWB: non-weightbearing; LCP: locking compression plate.

## Discussion

This case illustrates a fixation problem at the intersection of two well-described but rarely combined challenges: a comminuted fibular head avulsion and a retained ipsilateral tibial intramedullary nail. The central contribution is a reproducible hybrid construct - transosseous Krackow sutures through the biceps femoris, supplemental buttress with an LCP hook plate, and primary LCL repair - that addresses both the soft-tissue and bony components of the injury while routing around retained hardware.

The standard options each have a structural prerequisite this patient lacked. Cannulated or tibiofibular screw fixation requires a fragment large enough to capture and a trajectory unobstructed by existing hardware [7]. Hook plates applied to the proximal fibula restore the bony attachment - Kim et al. described a locking compression hook plate combined with whipstitch suture repair for a comminuted proximal fibular avulsion with LCL injury [8] - but screw purchase and trajectory remain constrained when a tibial nail and its interlocking screws occupy the proximal tibia. Suture anchor or transosseous repair addresses the soft-tissue avulsion but assumes intact bone for anchor seating or tunnel drilling [9]. Comminution alone has driven others to alternative constructs, such as the tension-slide technique reported by Cinats and Stone [10]. In this patient, the comminuted fragment could not hold a screw, and the retained tibial nail blocked the conventional transosseous drilling path. The hybrid solution addressed both problems sequentially: the Krackow-through-Beath-pin construct re-tensioned the biceps femoris through a distal anteromedial trajectory that avoided the nail, and the LCP hook plate - whose tines engaged the comminuted fragment without requiring screw purchase within it - provided supplemental buttress and compression. That varus stress gapping reduced from +3 to neutral after fixation supports the biomechanical adequacy of the construct, at least acutely.

The polytrauma context of this case warrants discussion. The patient sustained multiple concomitant injuries - a left first rib fracture, fractures of the sacrum, pubic root, and bilateral superior pubic rami, and retained metallic foreign bodies in the gastrointestinal tract - in addition to the open proximal tibial fracture requiring emergent intramedullary nailing and, identified after nailing, the fibular head avulsion with peroneal nerve palsy. Examination under anesthesia demonstrated a positive Lachman test consistent with a concurrent ACL injury, which was not addressed at this stage. This is consistent with a staged approach in a polytrauma patient: restoring the posterolateral corner first to prevent the varus stress that would jeopardize a future cruciate reconstruction [5]. At the time of this report, no decision had been made regarding staged ACL reconstruction; management of the ACL remained to be determined pending the patient's rehabilitation course and any residual symptomatic instability.

Combined bony and neurologic management is a second notable feature. Common peroneal nerve injury accompanies a substantial proportion of PLC and fibular head injuries - approximately one quarter of operative PLC injuries in one series [4] - yet the literature offering operative detail on simultaneous fixation and formal neurolysis in this setting is limited. In this case, the nerve was found in continuity with edema and swelling consistent with a neurapraxic or axonotmetic injury pattern. Neurolysis was performed to decompress the nerve and confirm continuity, with a plan for serial clinical surveillance and potential electromyography. Published outcomes after traumatic knee injury temper expectations: in a systematic review of peroneal nerve palsy following knee dislocation, only 38% of complete palsies achieved functional motor recovery, whereas most partial injuries recovered fully [12]. By the nine-week follow up visit, the palsy had resolved completely, with full motor and sensory recovery on clinical examination. Given complete recovery, EMG was not indicated. This course is consistent with the neurapraxic pattern suspected intraoperatively.

The use of an LCP hook plate at the proximal fibula merits comment. Although this implant family is most commonly used at the medial malleolus, it is designed for application across multiple anatomic sites. Its hook-and-plate geometry - sharp tines that capture a small fragment while the plate body provides buttress - proved well-suited to this anatomy, and the low-profile design minimized the risk of soft-tissue irritation over the fibular head. Repurposing an existing implant geometry for an unconventional location has established precedent in orthopedic practice, as recently reviewed for the proximal humeral internal locking system plate applied across the femur, tibia, clavicle, and other sites [13].

Limitations

This is a single case, and the follow-up interval is short: the most recent documented visit occurred at nine weeks, which is sufficient to demonstrate maintained fixation and early interval healing but not to establish union, durable stability, or final functional outcome. The common peroneal nerve palsy had resolved completely by the nine-week visit; however, longer-term follow-up would be needed to confirm durable neurologic recovery. The concurrent ACL tear was managed nonoperatively, and the patient declined further surgical intervention; longer-term follow-up would be needed to assess for any residual functional instability. The construct combines a site-adapted implant application with a patient-specific tunnel strategy, so generalizability is limited and longer-term durability is unknown. The polytrauma setting introduces confounders for rehabilitation compliance and functional outcome assessment. Nonetheless, the technique is reproducible with widely available implants and may be useful when comminution and retained hardware together defeat conventional fixation strategies.

## Conclusions

When fracture comminution precludes screw purchase and a retained tibial intramedullary nail obstructs conventional transosseous drilling paths, a hybrid construct combining transosseous Krackow sutures routed around the retained hardware with supplemental hook-plate buttress fixation and primary ligament repair offers a technically feasible salvage option using widely available implants. This approach may restore posterolateral corner stability and preserve the bony and soft-tissue attachments of the fibular head in settings where standard single-implant techniques are not applicable. At the last follow-up, the patient had regained the desired knee range of motion, advanced to weight-bearing as tolerated, and demonstrated maintained alignment with early radiographic healing changes and intact hardware. The common peroneal nerve palsy resolved completely, attributed to a combination of spontaneous recovery and intraoperative neurolysis. Limitations of this report include the short duration of follow-up, the absence of advanced imaging to confirm definitive osseous union, single-patient design, and the unresolved ACL tear. This technique may be considered when comminution and retained hardware preclude conventional fixation of fibular head avulsion fractures.
